# Oral S2-Ag85 DNA Vaccine Activated Intestinal Cell dsDNA and RNA Sensors to Promote the Presentation of Intestinal Antigen

**DOI:** 10.1155/2022/7200379

**Published:** 2022-04-13

**Authors:** Dang Sheng, Sui Xin, Wu Dongxing, Wen Shubo, Song Yang, Chen Zeliang, Zhai Jingbo

**Affiliations:** ^1^Innovative Institute of Zoonoses, Inner Mongolia Minzu University, Tongliao 028000, China; ^2^Brucellosis Prevention and Treatment Engineering Research Center of Inner Mongolia Autonomous Region, Tongliao 028000, China; ^3^Key Laboratory of Zoonose Prevention and Control at Universities of Inner Mongolia Autonomous Region, Tongliao 028000, China

## Abstract

**Objective:**

To explore the molecular mechanism by which oral S2-Ag85DNA vaccines present intestinal antigens. The oral S2-Ag85 vaccine has been shown to protect the human body and effectively improve the titration of the vaccine by acting on intestinal mucosa cells and enhancing their immunogenicity.

**Method:**

Mice were immunized with the recombinant S2-Ag85 vaccine, and antibody secretion was then detected in the intestinal tissue. The molecular mechanisms of *in vitro* detection sensor molecules RIG-1, Pol III, and related conductor transductor molecules DAI, STING, AIM2, IRF3, and IRF7 were determined by separating intestinal IEC, DC, and IELC cells.

**Results:**

The S2-Ag85A vaccine was effective in activating dsDNA and RNA transduction pathways in intestinal cells and improving intestinal antigen presentation in mice.

## 1. Introduction

Brucellosis is a zoonotic disease that affects humans worldwide [1]. Its main pathogenic mechanism is via interference with host cells to induce an inherent immune response, affecting the production of adaptive immune responses in the body [2]. When *Brucella* species enter the body, the host cells activate the body's inherent immunity and adaptive immunity by identifying receptors (PPR) and specific molecular structures in the pathogen, producing an immune response [3–5]. During the adaptive immune activation of the body against *Brucella*, IFN-*γ* is produced by CD4 T and CD8 T cells and activates macrophages, which release immunogenic substances to further resist the survival and replication of *Brucella* in cells [6]. However, *Brucella* can evade this adaptive immune response by the following mechanism: (1) the *Brucella* surface's lack of adaptive immunity can activate the substance to escape being damaged by the host's adaptive immune response [7]; (2) *Brucella* can reduce or hide its pathogen-related molecular patterns, evading macrophages and phagocytes with endosome mesh fusion by lipid rafts enabling further replication in body cells [8]. In general, after *Brucella* enters the body, the body cannot produce an effective immune response.

Antigen 85 is present in cell walls and culture filters in organisms such as *Bacillus tuberculosis* and BCG [9]. Antigen Ag85A has strong Th1 cell induction potential and can better induce immune responses by CD4 and CD8 T cells [10]. Numerous studies have shown that AG85A can stimulate the production of individual peripheral blood cells in patients with inoculated carcinoma or with binding diseases, improving the immunogenicity of patients [11, 12].

The oral S2 vaccine can protect against brucellosis by 40% to 60%, but the current immune dose of the oral S2 vaccine is 20 billion units [13–15], which increases the risk of animal vaccine poisoning. To improve the protection offered by the vaccine and overcome the disadvantage of using too large a dose, the S2-Ag85A oral DNA vaccine was developed. This uses the Ag85A antigen to increase the immunogenicity of the organism, thus increasing the potency of the vaccine. The basis on which the immune system generates an immune response is the efficient transduction of intracellular DNA transduction molecules. In the present study, the sensor molecules of the oral S2-Ag85A DNA vaccine and its mechanism of action in the intestine were investigated.

## 2. Materials and Methods

### 2.1. Mice

The C57BL/6 mice were 7–8 weeks old and consisted of 18 males and 18 females. The animals were purchased from Beijing Huafukang Biotechnology Co., Ltd.; the license number of the experimental unit was SCXK (Beijing) 2019-0008. Animal care and all the experiments were reviewed and approved by the Research Ethics Committee of Inner Mongolia University.

### 2.2. Vaccine

The S2 vaccine was purchased from Qilu Animal Health Co., Ltd., with approval number (2015) 150257011. The S2-Ag85A vaccine was developed independently by the laboratory.

Preparation of S2-ag85A vaccine: (1) Preparation of receptor state: *B. brucei* (OD600 = 0.20) completely cultured in TSA liquid medium was placed in an ice bath for 15 min, and the bacteriophage was collected by centrifugation at 4°C 4000 rpm/min for 5 min. Wash the bacterium twice with deionized water and then wash it once with 15% glycerol, dispense it by 100 *μ*L/tube, and store it at −80°C for backup. (2) Construction of CRISPR/Cas9 plasmids: determine the S2 genomic target sequence and design and synthesize five sgRNAs corresponding to 20 nt DNA oligonucleotide sequences in the target sequence and synthesize them, clone them into the CRISPR/Cas9 plasmids formed by PX330 plasmids, test the activity of the five CRISPR/Cas9 plasmids, respectively, and select the most active. The CRISPR/Cas9 plasmid with the highest activity was selected for subsequent cotransformation of sensory cells. Electrotransfer conditions (none point transfer): pulse voltage 1000 V; pulse wide 40 ms; pulse no. 1 pulse (translated with http://www.DeepL.com/Translator (free version)). The activity of 5 CrisPr/Cas9 plasmids was detected, and the most active Crispr/Cas9 plasmid was selected for subsequent coconversion sensitive cells. Electric turn conditions (NONE point transfer) are as follows: pulse voltage 1000 V, pulse wide 40 ms, and pulse no. 1 pulse. (3) Construction of targeting plasmid pTg2.0-Ag85A: the cDNA of Ag85A was cloned into PTg2.0 vector, and positive clone screening was performed using puromycin resistance. (4) Positive clones and detection: PCR (primer information) and double digestion (Afl II and EcoR I sites) were used to identify whether the S2 gene was inserted into the Ag85A gene.

### 2.3. Immunization and Grouping

C57BL/6 mice were randomly divided into three groups of 12 mice each: the S2-Ag85A group, S2 group, and normal control group. Each mouse was immunized three times by gavage of 0.2 mL of bacterial solution (6∗10^4^), with an interval of 10 days between the first two immunizations and 14 days between the second and third. The same method was used to gavage the S2 group with the bacteriological solution. Mice in the normal group were fed with the equivalent volume of saline.

### 2.4. Isolation and Culture of Intestinal Mucosal Epithelial Cells (IEC)

After the please enter test, the mice were carefully removed from the intestinal membrane under a microscope, transferred to a petri dish, and repeatedly cleaned with PBS to wash the mucus on the surface of the intestine contents and intestinal mucosa until the supernormal fluid was clear, and then washed several times with a serum-free DMEM-F12 culture base containing penicillin (300 U/mL) and streptomycin (0.3 g/L). The intestinal tube is first cut into 2 to 3 mm tissue fragments, the use of PBS cleaning tissue blocks 6 to 8 times, in the cut into 1 mm^3^-sized pieces. A mixture of 1 : 1 was added to the pancreatic enzyme, and type I collagen digestive fluid was 10 mL, placed vertically in a cell culture bottle of T25 on the oscillator at 37 degrees C oscillating digestion 20 min, and then placed under an inverted microscope to observe cell digestion in the digestive fluid. In case when there are more digested cell clusters in the digestive fluid, the digested cell solution 5 min is repeatedly blown by pipettes, and the blown cell solution is transferred to 15 mL centrifugal tube, 1000 r/min, and centrifugal 5 min. The supernatant was discarded and washed by readding PBS solution and repeatedly blowing for 5 min, followed by centrifugation at 1000 r/min for 5 min, and the supernatant was discarded. Using the medium resuspended precipitation, the cells were placed in a 24-well plate with 2% gelatin wrap and placed at 37 degrees C and 5% CO2 incubator culture.

### 2.5. Isolation and Culture of Intestinal Mucosal Lymphoid Dendritic Cells (DC)

Dendritic cells were isolated using the immunomagnetic bead method, and the intestinal mucosa lamina propria mononuclear cells (LPMC) were isolated. The isolated LPMC cells were cultured overnight, following which the cell suspension was washed twice with PBS and resuspended. Dead cells were removed by gradient centrifugation, and the filtrate was prepared into a cell suspension and counted. Cell sorting buffer (90 *μ*L) and CD11c antibody-coated magnetic beads (10 *μ*L) were added for every 107 cells, and the resulting suspension was mixed well and incubated on ice for 15 min. Precooling buffer (1.5 mL) was added, following which the suspension was centrifuged; the supernatant was removed, and the cells were resuspended in buffer (500 *μ*L). The resuspended cells were placed into a MACS sorting rack, and the cell suspension outflow was collected. After the liquid flow dried up, buffer (500 *μ*L) was added and rinsed three times, and the nonbound cell fraction of immunomagnetic beads was collected. The collected cells were denoted DC cells.

### 2.6. Isolation and Culture of Intestinal IEL Cells

The small intestine was turned over and rinsed twice using Hank's solution containing 2% double antibodies, then placed in a centrifuge tube containing trypsin/collagenase, and incubated for 40 min at 37°C. The tube was removed and shaken thoroughly, and the digest was filtered through a 300-mesh filter. The filtrate was centrifuged at 1,000 rpm for 1 min, and then, the supernatant was discarded, and the volume was adjusted to 5.4 mL by adding 1640 culture solution supplemented with 5% NCS. Then, 100% Percoll was added to make the volume up to 9 mL, and the resulting mixture was mixed thoroughly to form a 40% cell Percoll suspension. 70% Percoll (1 mL) was gently added to the bottom of the barrel capillary tube, forming a junction between the two layers of liquid and resulting in a Percoll gradient separation solution. This was centrifuged at 660 rpm at 25°C for 20 min, following which the cells at the interface of the 40% and 70% Percoll solutions were washed three times with 1640 medium containing 5% NCS. These cells were denoted LEL cells.

### 2.7. Enzyme-Linked Immunosorbent Assay

After the immunization process, the three groups of mice were uniformly dissected, and intestinal segments were retained. Intestinal segments of 10 mice from each group were taken, washed, ground, and analysed by indirect enzyme-linked immunosorbent assay (ELISA) for IgG (Abcam; ab151276), IgA (Abcam; ab157717), IgM (Abcam; ab215085), SIgA (Elabscience; E-E m1040c), IFN-*β* (Abcam; ab252363), IFN-*γ* (Abcam; ab239425), TNF-*α* (Abcam; ab208348), IL-1*β* (Abcam; ab222503), ICAM-1 (Wuhan Saipei; SP13706), MIP-1*α* (Abcam; ab155449), TLR4 (Wuhan Saipei; SP30149), and CXCL10 (Wuhan Saipei; SP13698) expression according to the manufacturer's instructions. Subsequently, the plates were placed in enzyme marker to read the OD value at the corresponding wavelength. Standard curves were plotted according to the OD values, and the corresponding concentration of each enzyme was found.

### 2.8. RT-PCR and Q-PCR

Total RNA was extracted from cells using TRIzol reagent (Invitrogen; 15596018) according to the kit instructions, and cDNA was obtained by reverse transcription. The RT-PCR kit (Invitrogen 12594100) was used to detect the relative expression levels of IFN-*α*, IL-6, TNF-*β*, and MCP-1 in IEC and DC cells. The relative expression levels of TECK, CD80, CD11, CD86, MCHII, CCL5, cAMP, cGAS, TBK1, NF-*κ*B, IRAK4, and MYD88 in IEC and DC cells were determined using *β*-actin as the internal reference, and the reaction system and conditions were as per the manufacturer's instructions. The 2^-△△^ct was used to calculate the relative expression levels of mRNA.

### 2.9. Western Blot

After the immunization process, the mice were dissected, and the intestinal segments were preserved for western blot assay. The concentration of extracted protein was determined by the BCA method, and the upper sample concentration was 20 *μ*g·well^–1^. The protein was separated by SDS-PAGE electrophoresis and transferred to PVDF membrane. First, antibody STING (1 : 1000), AIM2 (1 : 1000), DAI (1 : 10000), IRF3 (1 : 1000, Abcam, Cambridge, MA, USA), IRF7 (1 : 1000, Abcam, Cambridge, MA, USA) 4°C overnight. Horseradish peroxide binding secondary antibody (1 : 1000, AmyJet Scientific Inc., Wuhan, China), RT incubate for 1 hour and display the image with ECL chemiluminescence reagent. GADPH (1 : 1000, Abcam) used as loading control. Bio-Rad Gel Doc EZImager (Bio-Rad, California, USA) Develop.

### 2.10. Detection of T-Cell Subpopulations in IEL Cells

IEL cell suspension (l × l0^6^ mL^–1^) was centrifuged at 2,500 rpm for 20 minutes, and the supernatant was discarded. Cell suspension (4 mL) from each group of cells was washed once with PBS containing 2% calf serum to remove most of the remaining supernatant, leaving 30–40 *μ*L of liquid. The cells were suspended by flicking, and 20 *μ*L of labelled antibody was added. Three control tubes were incubated with monochrome fluorescent labelled antibody for 20–30 min. Cells were resuspended in the appropriate volume of wash solution. Flow cytometry analysis was performed to determine T-cell subsets.

### 2.11. Statistical Analysis

Results are expressed as mean ± SD. Data were analyzed using one-way analysis of variance (ANOVA) with the SPSS version 18.0 software (SPSS Inc., Chicago, IL, USA). *P* < 0.05 was considered to indicate statistically significant differences.

## 3. Results

### 3.1. Oral S2-Ag85 Vaccine Increases the Expression of IgG, IgM, and IgA in the Gut of Mice

**Figure 1:**
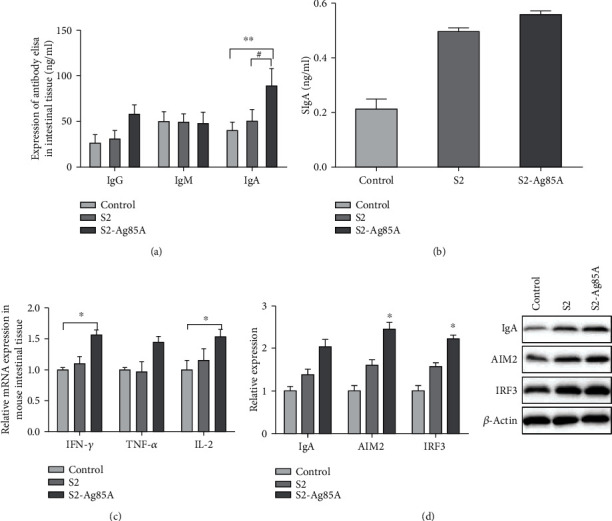
Oral S2-Ag85 vaccine increases the expression of IgG, IgM, and IgA in the gut of mice. (a) Oral S2-Ag85 vaccine increases the expression of IgG, IgM, and IgA in the gut of mice. (b) Content of IgG, IgM, and IgA in the intestine of mice in each group as determined by ELISA, compared with the control group. (c) Content of SIgA in the intestinal tissue of mice in the three groups. (d) Expression of IgA, AIM21, and IRF3 proteins in the intestinal tissue of mice in the three groups. ^∗^*P* < 0.05; ^∗∗^*P* = 0.01; compared with the S2 group #*P* < 0.05; ##*P* = 0.01.

To determine whether the oral S2-Ag85A DNA could increase intestinal antibody activity and content, mice were dissected, and intestinal segments were retained 3 weeks after the final immunization. ELISA was performed to detect IgG, IgM, IgA, and SIgA contents in mouse intestinal tissues, and western blot was performed to detect IgA, AIM2, and IRF3 in mouse intestinal tissues. The results are shown in Figure 1; compared with the normal control group, IgG, IgA, and SIgA contents in intestinal tissues were increased in the S2 vaccine and S2-Ag85A vaccine groups, with IgA expression in the S2-Ag85 group being significantly higher than that in the control and S2 groups (*P* < 0.05). The high expression of IgA, AIM2, and IRF3 proteins in the S2-Ag85A group indicated that S2-Ag85A could effectively increase the content of transduced molecular proteins in the intestinal tissue of mice and increase the secretion of antibodies in the intestinal tissue (Figure 1).

### 3.2. Changes of Antibodies and Interferons in IEC and DC Cells of Mice Induced by the S2-Ag85 Vaccine

**Figure 2:**
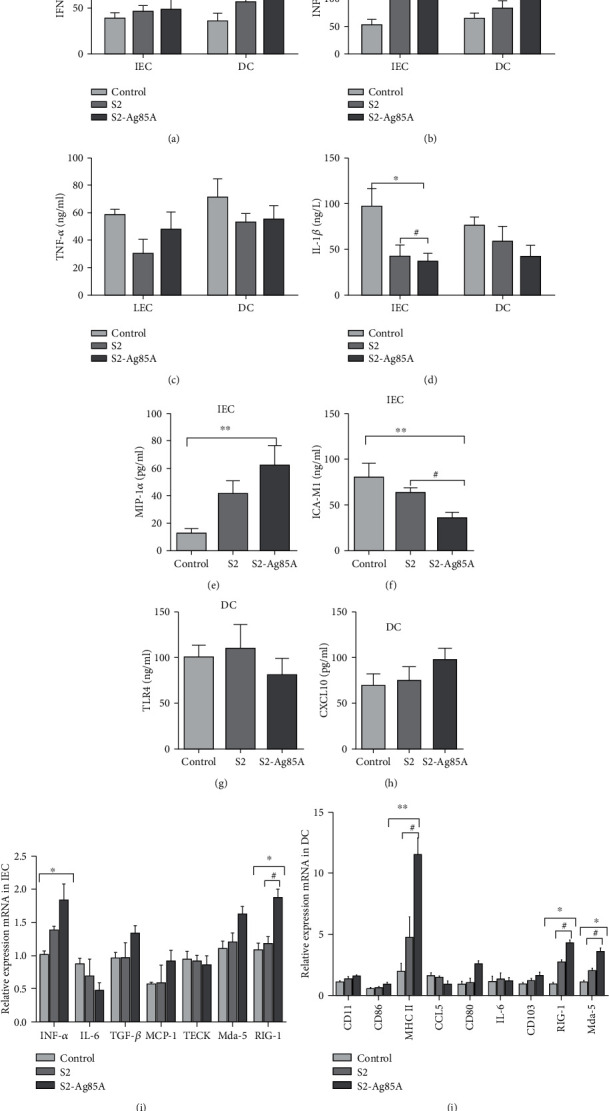
The S2-Ag85A vaccine promotes antibody secretion from IEC and DC cells to reduce the production of proinflammatory factors. ELISA to detect (a) IFN-*β* expression in IEC and DC cells, with significantly increased IFN-*β* expression in DC cells of the S2-Ag85A group (*P* < 0.05); (b) IFN-*γ* expression in IEC and DC cells, with significantly increased IFN-*γ* expression in DC cells of the S2-Ag85A group (*P* < 0.05); (c) IL-1*β* expression in IEC and DC cells, with significantly increased IL-1*β* expression in the S2-Ag85A group (*P* < 0.05); (d) IL-1*β* expression in IEC and DC cells, with significantly lower expression of IL-1*β* in the S2-Ag85A and S2 groups than the control group; (e) MIP-1*α* expression in IEC cells, with significantly increased expression in the S2-Ag85A group (*P* < 0.05); (f) ICA-M1 expression in IEC cells, with significantly lower expression in both the S2-Ag85A and S2 groups than in the control group (*P* < 0.05); (g) TLR4 expression in DC cells. (h) CXCL10 expression in DC cells. RT-PCR for (i) IFN-*α* (*P* < 0.05), IL-6, TGF-*β*, MCP-1, and TECK and (j) CD11, CD86, MCHII (*P* < 0.05), CCL5, CD80, and IL-6 in IEC cells.

To determine the effects of the S2-Ag85 vaccine on antibodies and interferons in mouse intestinal epithelial (IEC) cells and intestinal mucosal dendritic (DC) cells, intestinal epithelial cells and mucosal cells were isolated from the model animals for *in vitro* culture. ELISA was used to determine IL-1*β*, IFN-*β*, IFN-*γ*, TNF-*α*, ICAM-1, and MIP-1*α* contents (Figure 2). The IFN-*β* and IFN-*γ* contents in IEC and DC cells after stimulation with the S2 vaccine or S2-Ag85A vaccine were slightly higher than in the control group. The expression of ICA-M1 and MIP-1*α* in IEC cells after stimulation with S2-Ag85A was significantly higher than in the control group (*P* < 0.05), IL-1*β* content in IEC and DC cells was significantly lower than in the control group (*P* < 0.05), and IFN-*β* and IFN-*γ* contents in DC cells were significantly increased after S2-Ag85A stimulation (*P* < 0.05) (Figure 2).

The expression of IFN-*α*, IL-6, TGF-*β*, MCP-1, and TECK in IEC cells and CD11, CD86, MCH II, CCL5, CD80, IL-6, and CD103 in DC cells was determined by RT-PCR. In IEC cells, the expression of IFN-*α*, TGF-*β*, and MCP-1 was slightly increased, and the expression of IL-6 was decreased compared with the normal group, but the results were not statistically significant. After S2-Ag85A stimulation of DC cells, the expression of MCH II was significantly higher than in the control (*P* = 0.01) and S2 (*P* < 0.05) groups, and the expression of CD11, CD86, CCL5, CD80, and CD103 showed an increasing trend, while the expression of IL-6 decreased.

### 3.3. Recognition of IEC and DC Intracellularly Relevant Transduction Molecules by S2-Ag85 Oral Vaccine

**Figure 3:**
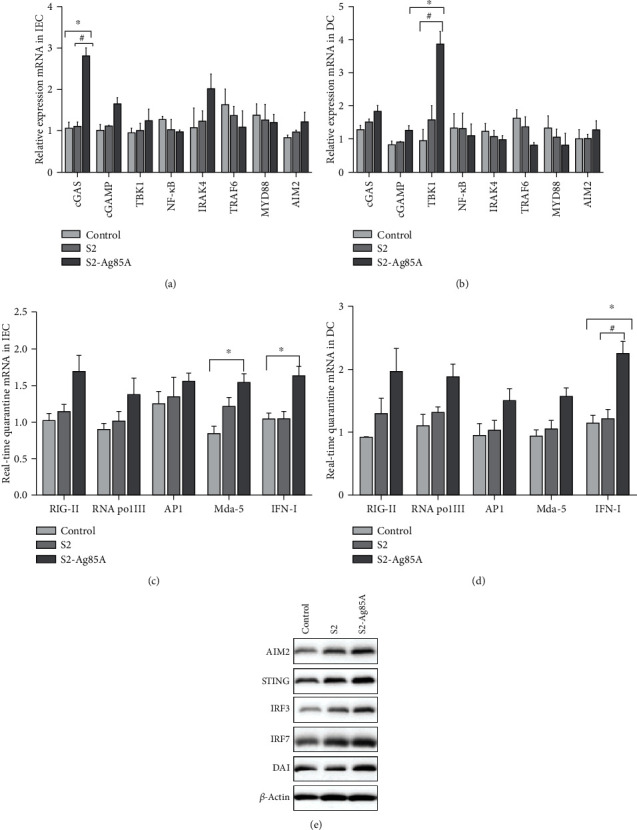
Effect of the S2-Ag85A vaccine on promoting dsDNA and RNA transmission pathways in IEC and DC cells. RT-PCR detection of (a) IEC cell sensor transduction molecules cGAS (*P* < 0.05), cGAMP, TBK1, NF-*κ*B, IRAK4, TRAF6, MYD88, and AIM2; (b) DC cell sensor transduction molecules cGAS (*P* < 0.05), cGAMP, TBK1 (*P* < 0.05), NF-*κ*B, IRAK4, TRAF6, MYD88, and AIM2. Q-PCR for (c) IEC cell sensor molecules RIG-I, RNA Pol III, and AP1; (d) DC cell sensor molecules RIG-I, RNA Pol III, and AP1. (e) Western blot for AIM2, STING, IRF3, IRF7, and DAI.

Ag85A is known to promote the presentation of antigens through dsDNA or RNA pathways. The results of the present study showed that, after stimulation with the S2-Ag85A vaccine, the expression of antibodies and interferons in mouse intestinal tissues as well as in IEC and SC cells was higher than in the control and S2 groups. To verify the mechanism of action of S2-Ag85A, the dsDNA and RNA signalling pathways in IEC and DC cells, respectively, were examined, and the relative expression of their related signalling molecules was detected using RT-PCR; the content of downstream proteins of related transduction molecules was determined by western blot, and the signalling molecules RIG-I, RNA Pol III, and AP1 were determined by Q-PCR. After receiving S2-Ag85A stimulation, the content of IEC cell signal transduction molecule cGAS was significantly higher than in the control and S2 groups, and cGAMP, TBK1, and IRAK4 showed an increasing trend, while NF-*κ*B and TRAF6 showed a decreasing trend (Figure 3). TBK1 expression was significantly higher in DC cells (*P* < 0.05), and IRAK4 and TRAF6 showed a decreasing trend. Western blot determined that the downstream protein expressions of STING, AIM2, IRF3, and IRF7 were all upregulated in IEC and DC cells after S2-Ag85A stimulation, with higher expression of these proteins than in the control group (Figure 3).

### 3.4. Detection of CD4+, CD8+, and + T Cells in IEL Cells

**Figure 4:**
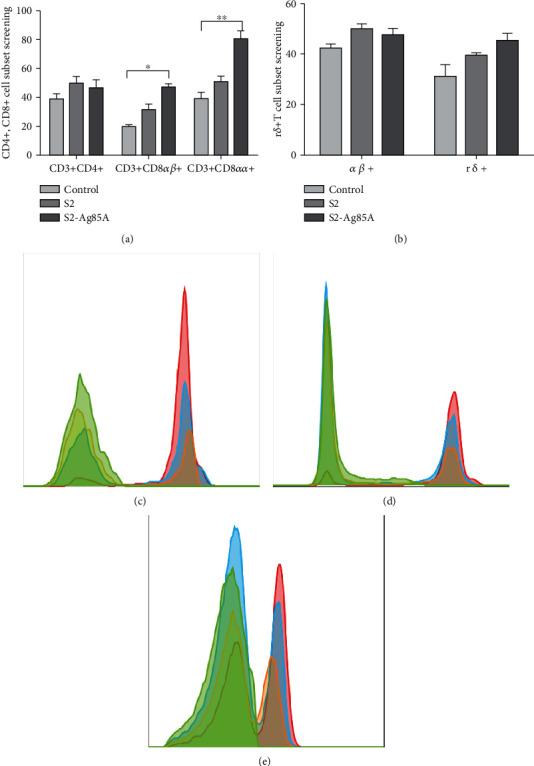
Proliferation of T cell subpopulations in IEL cells promoted by the S2-Ag85A vaccine. (a) CD4+ and CD8+ cell subpopulation screening: CD3 + CD8 + *αβ* and CD3 + CD8 + *αα* cell proliferation was significantly increased (*P* < 0.05). (b) +T cell subpopulation screening. (c) Flow assay CD8*α* + IEL subpopulation results; green, negative unstained; blue, normal control group; red, S2-Ag85A group. (d) Flow detection of CD8*β* + IEL subpopulation results; green, negative unstained; blue, normal control group; red, S2-Ag85A group. (e) Flow cytometric detection of TCR + T subpopulation results; green, negative unstained; blue, normal control group; red, S2-Ag85A group.

To further investigate the effect of S2-Ag85A oral vaccine on immune cells and their subpopulations, we examined the content of CD4+, CD8+, and medium *γδ*+T cell subpopulations in IEL cells. The T cell subpopulations were sorted using cell flow analysis. As shown in Figure 4, the CD8+ T cell subpopulation content in the S2-Ag85A group was significantly higher than that in the control group (*P* < 0.05), and there was no significant change in the +T content (Figure 4).

## 4. Discussion

Brucellosis is one of the most severe zoonotic diseases globally, causing abortion or infertility in cattle and sheep, leading to severe economic losses. The vaccines widely used for brucellosis in livestock are S19, Rev. I, and RB51, which can provide immunity to varying degrees, but the immunization results are unsatisfactory [16]. In 1952, the S2 vaccine was developed. S2 vaccines showed protection rates as high as oral vaccines; however, it requires a larger immunization dose, leading to greater toxicity risk after administration. Effective ways to reduce vaccine dosage and increase their effectiveness are constantly sought. A *Brucella* attack involves the bacteria invading the host mainly through the mucosa [17] and subsequently entering its cells to replicate, producing further pathogenic effects. So, mucosal immunity plays a vital role in brucellosis prevention.

Ag85A is an extracellular secreted antigen that promotes the mouse CD8+ cell proliferation, thereby stimulating the IFN-*γ* and TNF expression [18–20]. In this experiment, after the mice received the S2-Ag85A vaccine by gavage, the expression levels of IgG, IgA, IgM, and SIgA in the mouse intestine were detected by ELISA. The IgA content in the intestine of Ag85A mice was significantly higher than that control and S2 feeding alone groups, indicating that the S2-Ag85A oral DNA vaccine can significantly increase intestinal IgA secretion. IgA is mainly produced by lymphocytes on the intestinal mucosal tissue layer and plays an important role in the intestinal defense mechanism and mucosal immunity [21]. The increase in IgA secretion further increases the synthesis of SIgA in the intestine [22]. IgG functions in opsonization by phagocytic cells and the neutralization of toxins. Compared with the IgA-secreting cells, the number of IgG-secreting cells is significantly reduced. The amount of IgG secreted is also lower than IgA, and the amount of IgG secretion is closely related the digestive tract anatomy. This study focused on the small intestine of the mice, which is the main IgA secretion site. Therefore, although IgM secretion in the intestinal tract tends to increase, it is statistically different from the blank group. IgM is the main binding site of the antigen. When S2-Ag85A oral vaccine entered the intestinal tract, the protective effect on the intestine of mice was increased. As a part of the antigen receptor, the secretion of IgM is relatively reduced after the antibody response. This study showed a nonstatistically significant increasing trend in IgM compared to the blank and S2 groups, which may be related to the depletion of IgM-bound antibodies in the intestine. WB detected IgA, AIM2, and IRF3 in the intestinal tissues of the three groups of mice. The expression levels of the three proteins showed an upward trend compared to the control and S2 groups. The results are the same as previous studies, indicating that the S2-Ag85A vaccine can effectively promote the expression of immune proteins and enhance the protective ability of the intestinal mucosa.

After Brucella infects the body, it acts on the mucosa and reproduces and replicates in the mucosa. After intestinal mucosal epithelial cells (IEC) and dendritic cells (DC) are stimulated by S2-Ag85A, IFN-*β*, IFN-*γ*, IFN-*α*, TGF-*β*, and MCP, the secretion and expression of CXCL10, CD11, CD86, and MCHII increased in 1 and DC cells, and the expression of TNF-*α*, IL-1*β*, TLR4, TECK, and IL-6 decreased. It further shows that S2-Ag85A can induce IFN secretion by IEC cells and inhibit the expression of proinflammatory factors TNF-*α* and IL-6. In DC cells, S2-Ag85A can stimulate MHCII expression, which is only expressed in antigen-presenting cells [23] and further induces the production of CD11, CD86, and CD80, which in turn activates downstream chemokines [24].

Ag85A can increase immune function. After administration, Ag85A-containing vaccines produce Th1-like responses or stimulate the growth of T cells to further activate the body's immune response [25]. However, the immune response mechanism elicited by oral vaccines is not completely understood. This study showed that the S2-Ag85A vaccine stimulated IEL cells, the expression of CD8+ cells increased significantly (*P* < 0.05) but did not significantly change the number of CD4+ cells. S2-Ag85A oral vaccine can induce CD8 + T-mediated immune response. When S2-Ag85A stimulated IEC and DC cells, the cells' nondependent dsDNA receptor RNA Pol III content increased. The RNA Pol III activation mainly relied on RIG-I activation by STING to further induce the expression of IRF3, IRF7, and NF-*κ*B. S2-Ag85A is still effective for activation-dependent receptors. It promotes the expression of cGAS in cells, thereby better-activating cAMP, which activates downstream DAI molecules, STING protein, intestinal DNA, and RNA sensors, and promotes the presentation of the intestinal tract antigens.

This study shows that the S2-Ag85A oral vaccine induces Th1-type cytokine-mediated cellular immune response, enhances the immunogenicity of mouse intestinal mucosa, and is more conducive to optimizing the adequate vaccine titer. Therefore, S2-Ag85A can be used as an effective vaccine against brucellosis.

## Data Availability

No data were used to support this study.
